# Cholecystoduodenal Fistula, Gallstone Ileus, and Choledocholithiasis: A Case Report and Literature Review

**DOI:** 10.1002/ccr3.73641

**Published:** 2026-09-28

**Authors:** Jiaxu Ji, Yongping Huang, Dejun Tang, Wubulikasimu Wulamu

**Affiliations:** ^1^ School of Medicine The Chinese University of Hong Kong‐Shenzhen Shenzhen China; ^2^ Department of General Surgery The Second Affiliated Hospital, The Chinese University of Hong Kong‐Shenzhen & Longgang District People's Hospital of Shenzhen Shenzhen China

**Keywords:** case report, cholecystoduodenal fistula, choledocholithiasis, gallstone ileus, small bowel obstruction

## Abstract

Prompt diagnosis using CT is critical in gallstone ileus, as symptoms are often nonspecific. A combined surgical approach—comprising enterolithotomy, cholecystectomy, and fistula repair—effectively addresses both the obstruction and its underlying source, achieving favorable outcomes even in complex presentations.

## 1 Introduction

Gallstone ileus is a rare complication of cholelithiasis, in which a gallstone becomes lodged in the intestines, causing an obstruction. It accounts for 1%–4% of all intestinal obstructions and approximately 25% of obstructions in people over 65 years old [1]. Signs and symptoms are not specific, which makes early diagnosis difficult. The triad of small bowel obstruction, pneumobilia, and ectopic gallstone on a plain abdominal film is considered pathognomonic of gallstone ileus. However, it is only observed in 30%–35% of patients. Because it is a rare condition with high mortality and mainly affects older patients, it is necessary to choose safer treatment options. Existing treatments include antibiotics, fluid resuscitation, enterolithotomy, cholecystectomy, and closure of the fistula [2].

The pathogenesis of this disease involves pressure necrosis and erosion of the biliary tract wall into an adjacent structure, causing repeated inflammation and adherence, often with distal biliary tract obstruction [2]. Herein, we present the case of a 67‐year‐old man with gallstone ileus, cholecystoduodenal fistula, and choledocholithiasis, highlighting the diagnostic complexities and the successful implementation of a combined surgical approach.

## 2 Case Presentation

### 2.1 Case History and Examination

A 67‐year‐old male was admitted to the Department of Gastroenterology with 1 day of persistent colicky upper abdominal pain. The pain, which began on June 17, 2023, without an obvious trigger, was characterized by paroxysmal exacerbations and was accompanied by nausea and vomiting without relief after emesis. He reported no diarrhea, hematochezia, belching, or reflux. His medical history included hypertension and atrial fibrillation, managed with amlodipine besylate (5 mg daily), metoprolol succinate (47.5 mg daily), and atorvastatin calcium (20 mg nightly). He had no known allergies or other illnesses. Physical examination revealed a soft, flat abdomen with localized hepatic tenderness but no tenderness or rebound tenderness elsewhere. The liver and spleen were not palpable, Murphy's sign was negative, and shifting dullness was absent. Bowel sounds were 4/min.

### 2.2 Differential Diagnosis, Investigations, and Treatment

Emergency non‐contrast upper abdominal CT on June 18, 2023, demonstrated choledocholithiasis with acute cholangitis. The gallbladder was not clearly visualized (Figure 1a). Patchy gas densities in the left hepatic duct and a 10.2 mm hyperdense stone in the distal common bile duct suggested pneumobilia (Figure 1b) and choledocholithiasis (Figure 1c). Small bowel fluid with air‐fluid levels in the mid‐abdomen also indicated obstruction (Figure 1d). No ascites or retroperitoneal lymphadenopathy was seen. Based on the patient's presenting symptoms and physical signs, the initial diagnosis was choledocholithiasis with acute cholangitis. Gastrointestinal perforation and acute cholecystitis were initially excluded, given the absence of peritoneal signs, a negative Murphy's sign, and no evidence of intraperitoneal free gas or fluid collection on CT. The initial management plan in the Department of Gastroenterology comprised fasting, gastrointestinal decompression, antispasmodic therapy with phloroglucinol (40 mg once daily intravenously), anti‐infective therapy with cefoperazone/sulbactam (3 g every 8 h intravenously), acid suppression with esomeprazole (40 mg twice daily intravenously), fluid resuscitation, and supportive care. After 5 days of conservative treatment, the patient's symptoms persisted, with ongoing nausea, vomiting, and progressive signs of intestinal obstruction. Intravenous contrast‐enhanced (Iopromide) CT of the abdomen and pelvis was therefore performed on June 22, 2023, which demonstrated a cholecystoduodenal fistula (Figure 2a) complicated by cholecystitis, choledocholithiasis (Figure 2b, Figure 3), and gallstone ileus (Figure 2c, Figure 3). Based on the patient's clinical history, presenting symptoms, and physical signs, the diagnosis was revised to gallstone ileus with cholecystoduodenal fistula, complicated by choledocholithiasis and acute cholangitis. The abdominal pain was characterized by paroxysmal exacerbations, without acute‐onset severe or lancinating features. Furthermore, contrast‐enhanced CT showed no evidence of a “beak sign” or “whirl sign”, and no vascular filling defects were identified to suggest compromised mesenteric perfusion. Dynamic and vascular intestinal obstructions were therefore excluded. The patient was subsequently transferred to the Department of General Surgery for definitive surgical management on the same day. After transfer, the patient underwent standard preoperative workup, and no contraindications to surgery were found. Supportive and conservative management was maintained throughout the preoperative period. The surgery was performed on the second day following transfer (June 23, 2023) and consisted of laparoscopic enterolithotomy, conversion to open cholecystectomy, duodenal fistula repair, choledocholithotomy, choledochoscopy, and T‐tube drainage. The surgical procedure was carried out as follows: After establishing pneumoperitoneum via an infraumbilical incision, laparoscopic ports were placed at the subxiphoid area, the midpoint between the xiphoid and umbilicus, the midpoint between the pubic symphysis and umbilicus, and the right upper quadrant below the costal margin along the mid‐axillary line. Exploration revealed dense omental, duodenal, and colonic adhesions to the gallbladder fossa, along with extensive small bowel dilatation. Guided by preoperative imaging, a 2 × 2 cm stone was identified and clamped in the terminal ileum. With the patient in Trendelenburg position, an enterotomy was made over the stone, which was removed with aspiration of enteric contents (Figure 4). The intraoperative stone was not formally measured during surgery, and the stone was not sent to assess its composition. The enterotomy was closed with 3‐0 absorbable barbed sutures, reinforced with a seromuscular layer. The gallbladder was atrophic (2 × 3 cm) with thickened walls, and a cholecystoduodenal fistula was identified between the infundibulum and the duodenal bulb. Due to poor delineation of the common bile duct, conversion to open surgery was performed via a right subcostal curvilinear incision. Calot's triangle was dissected; the cystic artery was ligated and divided, and the cystic duct was traced and divided after confirming bile flow. A 2‐cm choledochotomy was made between stay sutures, through which a choledochoscope was inserted. A 1 × 1 cm stone was retrieved from the distal common bile duct using a basket, and exploration of the intrahepatic ducts was negative for residual stones. The duodenal fistula was repaired primarily with full‐thickness sutures and seromuscular reinforcement. A 16‐French T‐tube was placed, and the choledochotomy was closed with absorbable sutures, with a negative leak test. After irrigation, hemostasis, and placement of a subhepatic drain, the abdomen was closed. Estimated blood loss was 200 mL, and the patient was transferred to the ward in stable condition.

**FIGURE 1:**
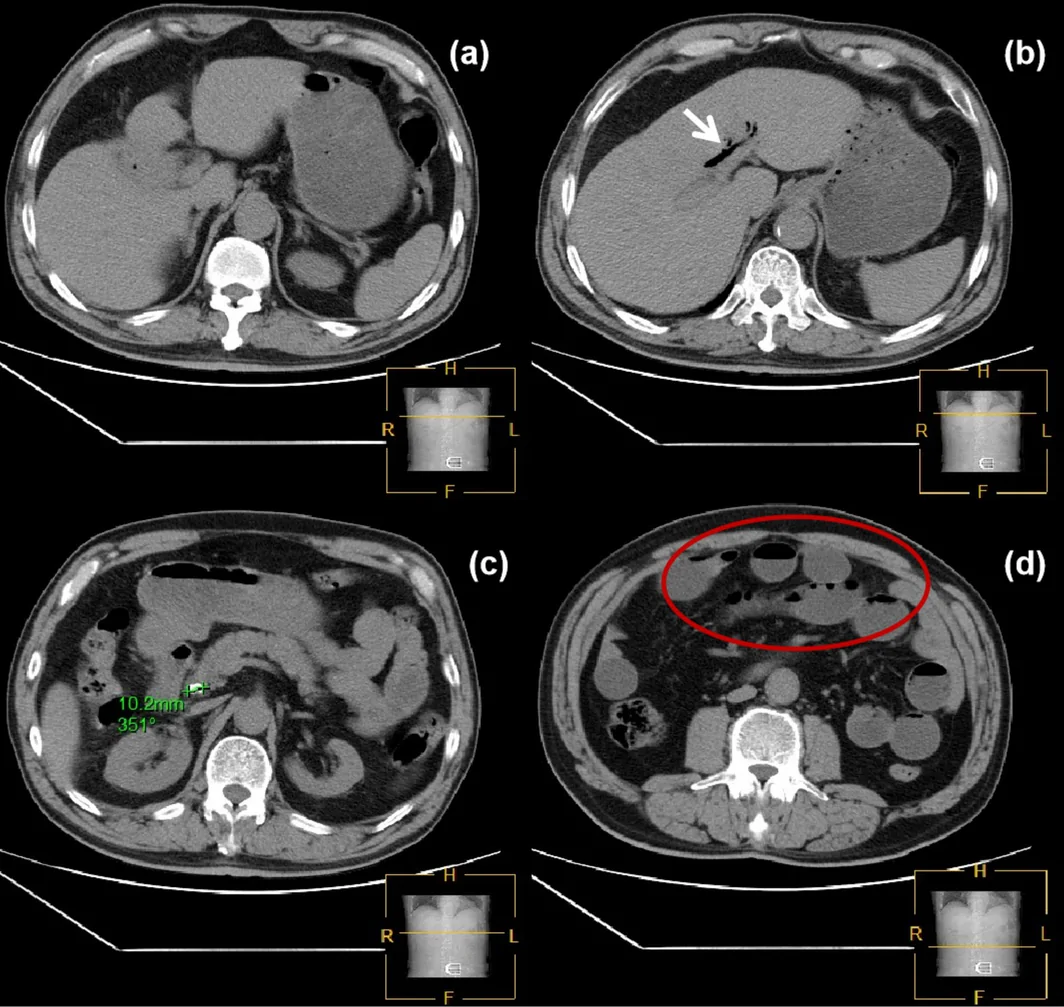
Non‐contrast axial CT of the upper abdomen. (a) The gallbladder is not clearly visualized. (b) Patchy gas densities within the left hepatic duct indicate pneumobilia (white arrow). (c) A 10.2 mm hyperdense stone is visible in the distal common bile duct, consistent with choledocholithiasis. (d) Small bowel dilatation with fluid accumulation and air‐fluid levels in the mid‐abdomen is indicative of small bowel obstruction (red circle).

**FIGURE 2:**
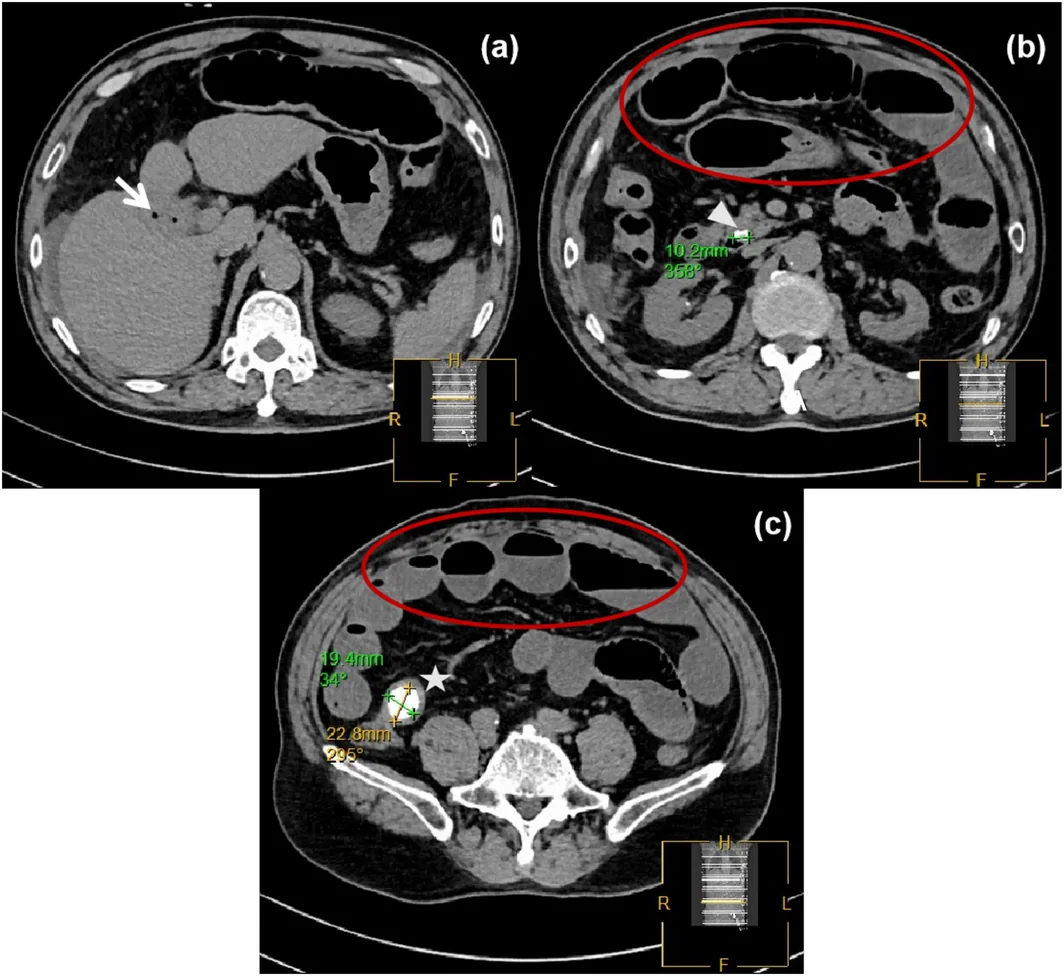
Non‐contrast axial CT of the abdomen and pelvis. (a) Pneumobilia (white arrow) indicative of a cholecystoduodenal fistula. (b) A 10.2 mm hyperdense stone in the distal common bile duct (white arrowhead) consistent with choledocholithiasis, with associated dilatation of the proximal small bowel (red circle). (c) A 22.8 mm × 19.4 mm intraluminal gallstone in the distal ileum (white star) causing small bowel obstruction, with prominent air‐fluid levels (red circle).

**FIGURE 3:**
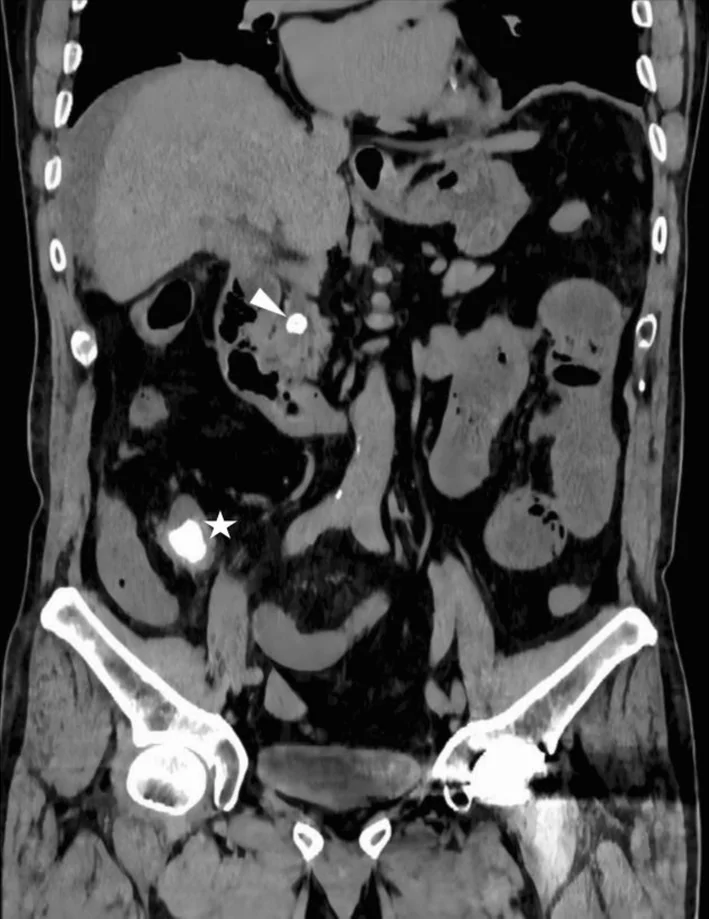
Non‐contrast coronal CT of the abdomen and pelvis. A hyperdense stone in the distal common bile duct (white arrowhead) consistent with choledocholithiasis and an intraluminal gallstone in the distal ileum (white star).

**FIGURE 4:**
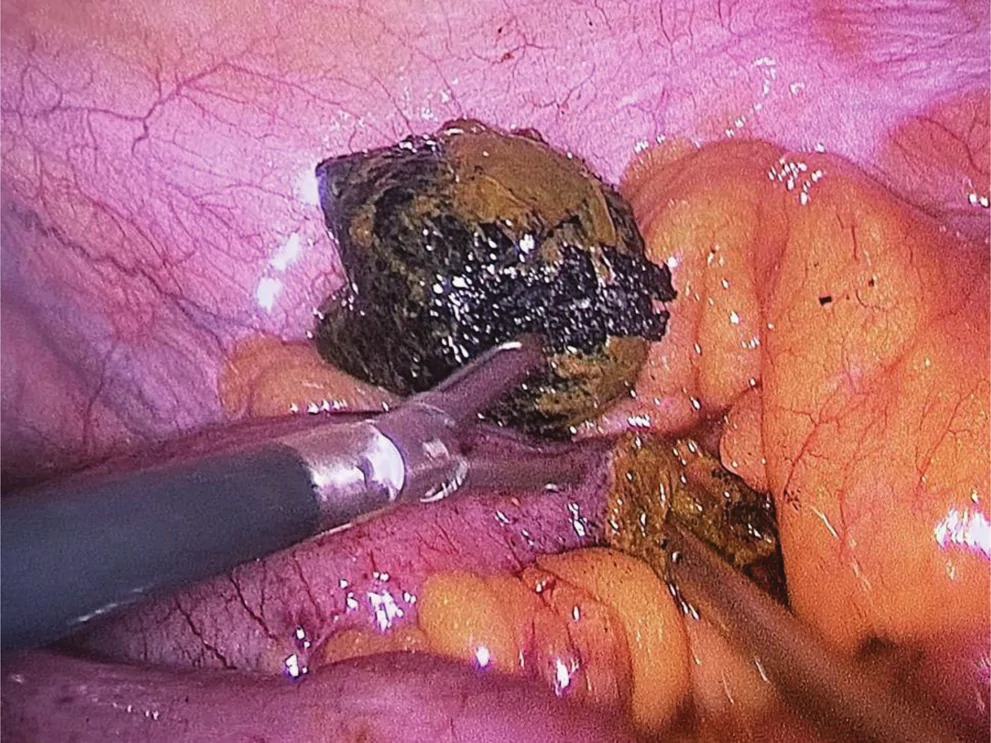
Intraoperative view demonstrating impacted stone (in black) in the ileum.

### 2.3 Outcome and Follow‐Up

Postoperatively, the patient continued to receive intravenous ceftazidime (2 g twice daily) for anti‐infective coverage, along with nasogastric decompression, acid suppression, analgesia, nutritional support, and symptomatic care. A follow‐up non‐contrast CT of the abdomen and pelvis was obtained on postoperative day 4 (June 27, 2023), which confirmed complete clearance of all stones and resolution of the small bowel obstruction (Figures 5 and 6). On June 28, 2023, the nasogastric tube was removed after the patient passed flatus, and oral intake was initiated with water only. The following day, bowel function returned, and the diet was advanced to a liquid diet and then gradually to a regular diet, with corresponding progressive resolution of abdominal symptoms. On postoperative day 6 (June 29, 2023), wound exudate was noted, and a secretion culture was sent for suspected wound infection. A wound infection was confirmed as a postoperative complication. Consequently, daily dressing changes were initiated, and the incision gradually healed. The intra‐abdominal drainage tube was removed on July 3, 2023. T‐tube cholangiography was performed on July 11, 2023, demonstrating clear and unobstructed intrahepatic and extrahepatic bile ducts (Figure 7). The T‐tube was subsequently removed on July 18, 2023. The patient was discharged on postoperative day 29 (July 22, 2023). At 1‐week and 1‐month follow‐up visits after discharge, the patient remained asymptomatic and had recovered well, with no complications. There were no readmissions, recurrent biliary symptoms, bowel obstruction, or mortality during the follow‐up period.

**FIGURE 5:**
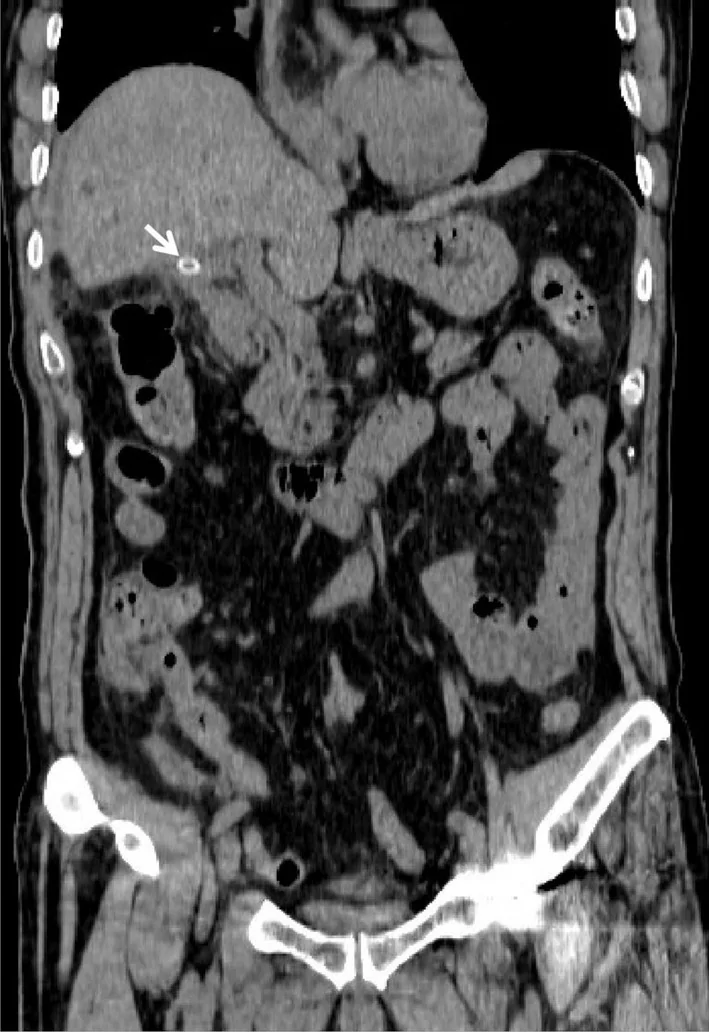
Post‐operative CT scan in the coronal plane revealing the absence of both common bile duct and intestinal stones. An upper intra‐abdominal drainage tube (arrow) was placed.

**FIGURE 6:**
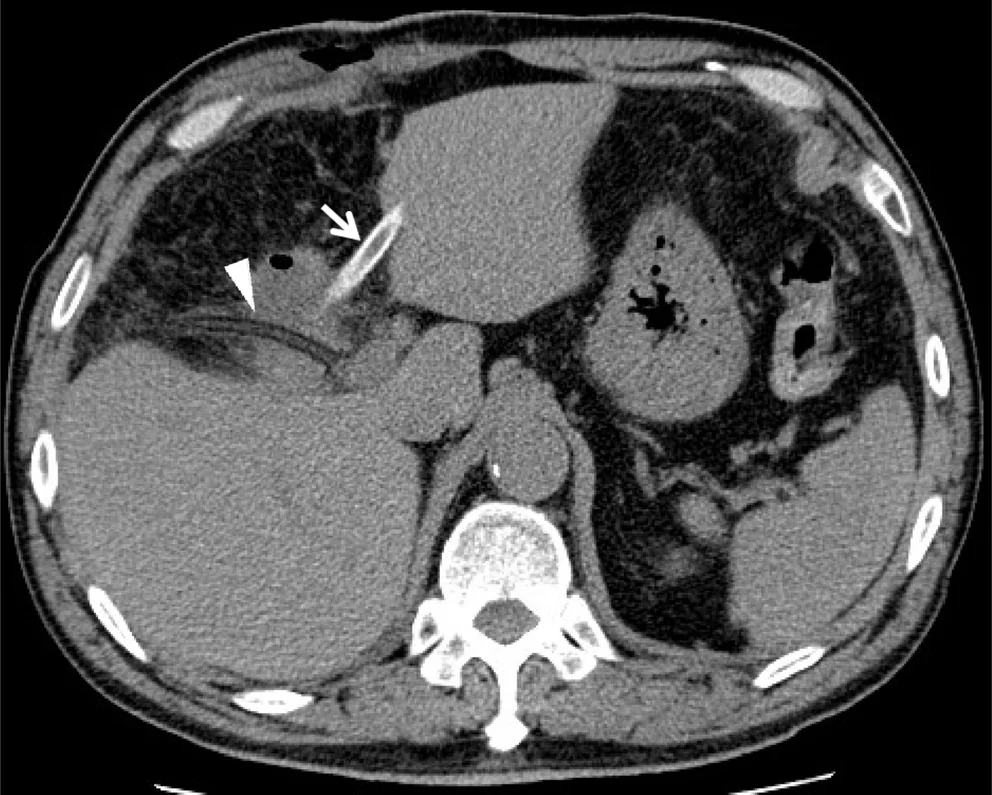
Post‐operative CT scan in the axial plane revealing that the upper intra‐abdominal drainage tube (arrow) and T‐tube (arrow head) were placed.

**FIGURE 7:**
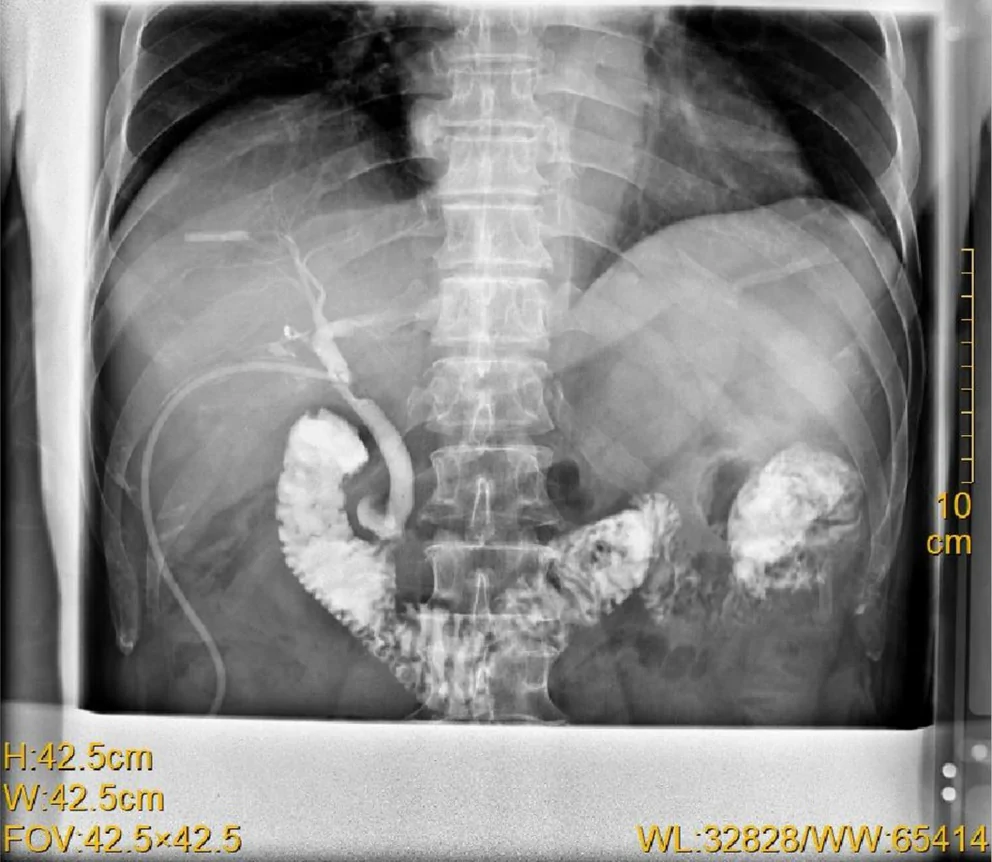
Post‐operative T‐tube cholangiography demonstrated clear and unobstructed intrahepatic and extrahepatic bile ducts.

## 3 Discussion

Gallstone ileus, a rare condition complicating 0.3%–0.5% of cases of cholelithiasis with a female‐to‐male ratio of 3.5 to 1, was first described by Bartholin in 1654 during an autopsy [2, 3]. After the formation of a cholecystoduodenal fistula, the gallstone can enter the bowel cavity through the fistula and become lodged, causing an obstruction. The most common fistula is cholecystoduodenal fistula (68%, *n* = 25), followed by cholecystcolonic fistula (5%) and cholecystduodenocolonic fistula (5%) [4]. The most common site of obstruction is the ileum (60.5% of cases), due to its narrow lumen diameter and less active peristalsis. The second common site is the jejunum (16.1%), followed by the stomach (14.2%), the colon (4.1%), and the duodenum (3.5%). It can also be passed spontaneously (1.3%). Gallstones larger than 2 cm are more likely to cause obstruction [4]. In our case, the presence of choledocholithiasis with acute cholangitis simultaneously in the same patient is exceptionally rare, making this case more noteworthy and challenging (Table 1).

**TABLE 1:** Rigler triad and findings in the present case.

Rigler triad	Classic findings	Findings in this case
Small bowel obstruction	Dilated small bowel loops with air‐fluid levels	Air‐fluid levels in small bowel (present)
Pneumobilia	Gas within the bile ducts	Patchy density shadows in the left hepatic duct (present)
Ectopic gallstone	Stone visible in the intestinal lumen	Approximately 2 × 2 cm stone impacted in the terminal ileum (present)

Because the symptoms of gallstone ileus are non‐specific, the diagnosis can be delayed by days [5]. Cholelithiasis patients associated with nausea, vomiting, and symptoms related to intestinal obstruction should be monitored more carefully. Ultrasound, CT, and MRI provide high early diagnosis rates. For CT, the overall sensitivity is 93%, the specificity is 100%, and the diagnostic accuracy is 99% [6]. For those older patients with suspicious symptoms, imaging examinations should be performed promptly, especially when their symptoms show some changes. In this patient, the initial computed tomography (CT) scan was limited to the upper abdomen and failed to visualize the distal ileal gallstone. Consequently, Rigler's triad was incomplete at that stage, which contributed to the delayed diagnosis of gallstone ileus. As a result, gallstone ileus and cholecystoduodenal fistula were diagnosed by a CT scan taken for transfer treatment.

Surgical interventions for gallstone ileus are mainly enterolithotomy alone, entero‐lithotomy with cholecystectomy, and enterolithotomy with fistula closure. However, there is currently no consensus regarding the best surgical approach in terms of mortality, morbidity, and operative outcomes. It is worth noting that combined enterolithotomy and cholecystectomy show a significant reduction in mortality (OR: 2.39 [95% CI: 1.87, 3.04], *I* ^2^ = 33%) compared to enterolithotomy alone (OR: 3.09 [95% CI: 1.36, 7.02], *I* ^2^ = 69%) [7]. The decision to perform a one‐stage definitive procedure rather than enterolithotomy alone was based on the patient's favorable preoperative status and the presence of concurrent biliary pathology. Recent evidence suggests that while enterolithotomy remains the mainstay of treatment for gallstone ileus, cholecystectomy and fistula repair can be performed in a single session in patients whose general condition is not critical [8]. Furthermore, a systematic review of colonic gallstone ileus found that the cholecystectomy rate was 46.7%, with 25% performed during the initial procedure and 21.7% as a separate procedure, indicating that definitive biliary surgery is not universally performed but is selected based on patient factors and clinical stability [9]. In our case, the procedure was converted from a laparoscopic to an open approach intraoperatively due to dense adhesions and distorted local anatomy that precluded safe laparoscopic dissection. However, this conversion did not alter the planned definitive management; instead, the open approach provided optimal exposure and tactile feedback to safely perform the cholecystectomy, fistula repair, and biliary exploration. In addition to surgical intervention, antibiotic therapy played a critical role in this patient's management. The patient initially received empirical therapy with cefoperazone/sulbactam for acute cholangitis, in accordance with the Tokyo Guidelines and local institutional susceptibility patterns. Following definitive surgical source control, the regimen was de‐escalated to intravenous ceftazidime to provide targeted coverage against common biliary pathogens ( *E. coli* , Klebsiella spp., and Pseudomonas spp.) while avoiding unnecessary broad‐spectrum β‐lactamase inhibitor coverage in the absence of multidrug‐resistant organisms.

Robotic management of cholecystoduodenal fistula is a new method that is used on patients who cannot tolerate open surgery or laparoscopic surgery. A 62‐year‐old woman with chronic cholangitis attributed to cholecystoduodenal fistula was reported to have undergone successful robotic cholecystectomy and fistula closure, and the symptoms subsided with no complications [10]. Another reported case is an 86‐year‐old female with an independent functional status and a history of medically‐managed cholecystitis. She underwent robotic‐assisted cholecystectomy and cholecystoduodenal closure. Her post‐operative recovery was also uneventful [11]. These two cases highlight the potential of robotic surgery to manage patients who refuse or cannot tolerate conventional surgery. However, due to the limited number of reported cases, robotic surgery should only be considered as a last resort option. Furthermore, in a meta‐analysis [12], researchers compared six postoperative outcomes of laparoscopic cholecystectomy (LC) and robotic cholecystectomy (RC) in 1013 patients. A random‐effect model analysis revealed that RC failed to prove any clinical advantage over LC for postoperative outcomes, including longer duration of operation. Moreover LC was more cost‐effective. The duration of operation was shorter in the LC group (mean difference: −10.23, 95% CI: −16.23 to −4.22, *Z* = 3.34, *p* = 0.0008) with moderate heterogeneity. The incidence of bile leak (odds ratio: 3.34, 95% CI: 0.85–13.03, *Z* = 1.73, *p* = 0.08) and postoperative complications (odds ratio: 1.49, 95% CI: 0.50–4.46, *Z* = 0.72, *p* = 0.47) were statistically similar between the two groups. LC also had a reduced cost (standardized mean difference: −7.42, 95% CI: −13.10 to −1.74, Z = 2.56, *p* = 0.01) with significant heterogeneity [13].

The techniques employed in the management of this case are standard clinical procedures. However, given that such rare conditions often present with atypical symptoms and signs, this case underscores the importance of vigilant clinical monitoring and prompt completion of relevant examinations—particularly contrast‐enhanced abdominopelvic CT—when symptoms evolve or when the initial therapeutic regimen proves ineffective. Surgical management, including enterolithotomy, cholecystectomy, and fistula closure, may be performed via laparoscopy or open surgery. In patients with concurrent biliary pathology, as in our case, combining existing surgical approaches to achieve radical treatment can yield satisfactory outcomes.

Several limitations of this case should be acknowledged. Because microbiological cultures were not obtained prior to antibiotic initiation, antibiotic treatment was empirical; the intraoperative stone was not formally measured, and stone composition was not assessed; and the current follow‐up duration (1 week and 1 month after discharge) is relatively short.

## Author Contributions

**Jiaxu Ji:** data curation, writing – original draft. **Yongping Huang:** formal analysis. **Dejun Tang:** formal analysis. **Wubulikasimu Wulamu:** conceptualization, supervision, writing – review and editing.

## Funding

The authors have nothing to report.

## Ethics Statement

The authors have nothing to report.

## Consent

Written informed consent from the patient was obtained for publication according to journal guidelines.

## Conflicts of Interest

The authors declare no conflicts of interest.

## Data Availability

All data supporting the findings of this case report are included in this article. Further details are available from the corresponding author upon reasonable request.
